# Effect of novel short‐arm human centrifugation‐induced gravitational gradients upon cardiovascular responses, cerebral perfusion and g‐tolerance

**DOI:** 10.1113/JP273615

**Published:** 2020-08-19

**Authors:** Charles Laing, David A. Green, Edwin Mulder, Helmut Hinghofer‐Szalkay, Andrew P. Blaber, Joern Rittweger, Nandu Goswami

**Affiliations:** ^1^ Institute of Aerospace Medicine German Aerospace Center (DLR) Cologne Germany; ^2^ King's College London Centre for Human and Applied Physiological Sciences (CHAPS) London UK; ^3^ Space Medicine Team HRE‐OM, European Astronaut Centre, European Space Agency Cologne Germany; ^4^ KBR Wyle Laboratories GmbH Cologne Germany; ^5^ Gravitational Physiology and Medicine Research Unit, Division of Physiology Medical University of Graz Austria; ^6^ Biomedical Physiology and Kinesiology Simon Fraser University Burnaby BC Canada; ^7^ Department of Paediatrics and Adolescent Medicine University of Cologne Cologne Germany

**Keywords:** artificial gravity, cardiovascular physiology, centrifuge, countermeasure, exercise physiology, short‐arm human centrifuge, spaceflight

## Abstract

**Key points:**

- The aim of this study was to determine the effect of rotational axis position (RAP and thus g‐gradient) during short‐arm human centrifugation (SAHC) upon cardiovascular responses, cerebral perfusion and g‐tolerance.
- In 10 male and 10 female participants, 10 min passive SAHC runs were performed with the RAP above the head (P1), at the apex of the head (P2), or at heart level (P3), with foot‐level Gz at 1.0 *g*, 1.7 *g* and 2.4 *g*.
- We hypothesized that movement of the RAP from above the head (the conventional position) towards the heart might reduce central hypovolaemia, limit cardiovascular responses, aid cerebral perfusion, and thus promote g‐tolerance.
- Moving the RAP footward towards the heart decreased the cerebral tissue saturation index, calf circumference and heart rate responses to SAHC, thereby promoting g‐tolerance.
- Our results also suggest that RAP, and thus g‐gradient, warrants further investigation as it may support use as a holistic spaceflight countermeasure.

**Abstract:**

Artificial gravity (AG) through short‐arm human centrifugation (SAHC) has been proposed as a holistic spaceflight countermeasure. Movement of the rotational axis position (RAP) from above the head towards the heart may reduce central hypovolaemia, aid cerebral perfusion, and thus promote g‐tolerance. This study determined the effect of RAP upon cardiovascular responses, peripheral blood displacement (i.e. central hypovolaemia), cerebral perfusion and g‐tolerance, and their inter‐relationships. Twenty (10 male) healthy participants (26.2 ± 4.0 years) underwent nine (following a familiarization run) randomized 10 min passive SAHC runs with RAP set above the head (P1), at the apex of the head (P2), or at heart level (P3) with foot‐level Gz at 1.0 *g*, 1.7 *g* and 2.4 *g*. Cerebral tissue saturation index (cTSI, cerebral perfusion surrogate), calf circumference (CC, central hypovolaemia), heart rate (HR) and digital heart‐level mean arterial blood pressure (MAP) were continuously recorded, in addition to incidence of pre‐syncopal symptoms (PSS). ΔCC and ΔHR increases were attenuated from P1 to P3 (ΔCC: 5.46 ± 0.54 mm to 2.23 ± 0.42 mm; ΔHR: 50 ± 4 bpm to 8 ± 2 bpm, *P* < 0.05). In addition, ΔcTSI decrements were also attenuated (ΔcTSI: −2.85 ± 0.48% to −0.95 ± 0.34%, *P* < 0.05) and PSS incidence lower in P3 than P1 (*P* < 0.05). A positive linear relationship was observed between ΔCC and ΔHR with increasing +Gz, and a negative relationship between ΔCC and ΔcTSI, both independent of RAP. Our data suggest that movement of RAP towards the heart (reduced g‐gradient), independent of foot‐level Gz, leads to improved g‐tolerance. Further investigations are required to assess the effect of differential baroreceptor feedback (i.e. aortic–carotid g‐gradient).

## Key points

- The aim of this study was to determine the effect of rotational axis position (RAP and thus g‐gradient) during short‐arm human centrifugation (SAHC) upon cardiovascular responses, cerebral perfusion and g‐tolerance.
- In 10 male and 10 female participants, 10 min passive SAHC runs were performed with the RAP above the head (P1), at the apex of the head (P2), or at heart level (P3), with foot‐level Gz at 1.0 *g*, 1.7 *g* and 2.4 *g*.
- We hypothesized that movement of the RAP from above the head (the conventional position) towards the heart might reduce central hypovolaemia, limit cardiovascular responses, aid cerebral perfusion, and thus promote g‐tolerance.
- Moving the RAP footward towards the heart decreased the cerebral tissue saturation index, calf circumference and heart rate responses to SAHC, thereby promoting g‐tolerance.
- Our results also suggest that RAP, and thus g‐gradient, warrants further investigation as it may support use as a holistic spaceflight countermeasure.

## Introduction

Immediately upon standing from the supine position, blood pressure remains unaltered at certain locations within the arterial, venous and other fluid‐filled compartments of the body, termed hydrostatic indifference points (HIPs, Kirsch *et al*. 1982). The locations of the HIPs depends on a number of factors, including filling volumes and sympathetic tone, yet the arterial supine‐to‐upright HIP has been localized at heart (aortic arch) level, whereas the venous supine‐to‐upright HIP is several centimetres below the apex of the diaphragm, i.e. liver level (Hinghofer‐Szalkay, 2011). As a result, receptors located at an HIP would not sense any pressure changes upon standing; in the arterial system, this applies to the aortic baroreceptors, which therefore convey information on systemic blood pressure undisturbed by the effects of postural changes. In contrast, carotid baroreceptors are positioned so that they can serve as the sentinels of cerebral perfusion (Mancia *et al*. 1984). Thus, with standing, carotid baroreceptors experience reduced vessel‐wall stress and consequently exert positive chronotropic and inotropic effects upon the heart. Similarly, cardiopulmonary (‘volume’) receptors that are above the venous HIP level sense a pressure drop with supine‐to‐upright positional changes, and therefore are also able to initiate cardiovascular responses (Hainsworth, 2014).

Exposure to microgravity induces a number of deleterious effects on humans including muscle atrophy, loss of bone mineral density and cardiorespiratory deconditioning including orthostatic intolerance (OI) (see Hargens & Richardson, 2009; Blaber *et al*. 2011; Goswami *et al*. 2012, 2013; Goswami, 2017). In addition to microgravity‐induced unloading, there is a headward fluid shift away from the lower limbs following the loss of the hydrostatic pressure gradient (Thornton & Hoffler, 1977). Long‐arm human centrifugation (LAHC) and high‐performance aircraft g‐exposure is associated with relatively dose‐dependent effects of +Gz upon the cardiovascular system and g‐tolerance. G‐tolerance is limited by an individual's ability to maintain cerebral perfusion which is impaired despite there being no significant g‐gradient (i.e. almost constant gravitational force is experienced at the head and feet, Scott *et al*. 2007). Repeated exposure to LAHC can improve orthostatic tolerance (Scott *et al*. 2013), but is impractical and thus unlikely to be employed during spaceflight (Clément & Pavy‐Le Traon, 2004).

However, recent research on Earth has shown that periodically shifting central blood volume to the periphery, through provision of artificial gravity (AG) of +2.4 Gz via short‐arm human centrifuge (SAHC) leads to significant improvements in subsequent standing orthostatic tolerance (Goswami *et al*. 2015*b*). SAHC is significantly more practical but leads to a g‐gradient where a substantially greater +Gz is experienced at the feet than the head (Clement & Pavy‐Le Traon, 2004). As a result, SAHC is associated with central hypovolaemia and syncope (Evans *et al*. 2015; Goswami *et al*. 2015*b*; White *et al*. 2019), which is a potentially critical situation should it occur in space. Whilst the AG required to ameliorate muscle atrophy, loss of bone mineral density and cardiorespiratory deconditioning is currently unknown, research has been instigated in order to optimize the AG ‘dose’ (i.e. duration and magnitude of exposure in terms of OI). However, to our knowledge no research has characterized the effects of varying the g‐gradient.

**Figure 1:**
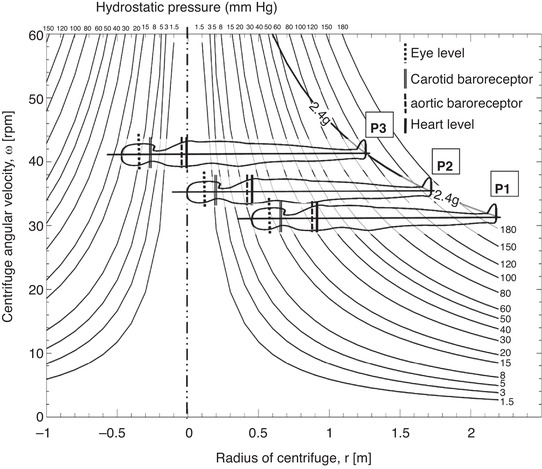
Hydrostatic pressure as a function of centrifuge radius and angular velocity A standard 1.73 m tall male participant is represented with the feet placed at an acceleration of 2.4 *g* (drawn as a function of angular velocity and centrifuge radius). Anthropometric positioning of eye level, carotid and aortic baroreceptors and heart levels are indicated for each of the three centrifuge positions P1, P2 and P3. For a description of pressure calculations see Table 5.

We hypothesize that the physiological responses to SAHC‐induced AG will differ between rotational axis position (RAP) since the hydrostatic pressure rises continually from head to foot when the rotation axis occurs above the head outside the body, but rises in both directions away from the rotation axis when it occurs within the body (Fig. 1). As a result, an RAP shift from above the head to heart level during SAHC, with a fixed g‐level at the feet, may improve g‐tolerance through attenuation of central hypovolaemia and impairment of cerebral perfusion. Furthermore, RAP – and thus the g‐gradient – will modulate the hydrostatic gradient between the carotid and aortic baroreceptors and thus cardiac filling, which should in turn affect the cardiovascular response to SAHC at a given AG, which may modulate g‐tolerance.

Thus the aim of the study was to investigate the effect of three standardised RAPs and the head‐to‐heart and heart‐to‐foot hydrostatic component (g‐gradient) of blood pressure upon the cardiovascular responses, peripheral blood displacement (i.e. central hypovolaemia), cerebral perfusion and g‐tolerance, in addition to the relationship between peripheral blood displacement, and both heart rate (HR) and cerebral perfusion with respect to g and RAP.

## Methods

### Ethical approval

The study conformed to the *Declaration of Helsinki*, including registration with the German Clinical Trial Registry (DRKS), and was approved by the Ethics Committee of the North Rhine Medical Association in Düsseldorf, Germany (Ref: 2015381). All participants gave written informed consent to participate in the study.

### Study design and participants

Twenty (10 male) healthy normotensive, non‐smoking participants (mean ± SD age: 26.2 ± 4.0 years; height: 1.73 ± 0.08 m; and BMI: 22.9 ± 1.7 kg m^−2^; Table 1) took part in the study.

**Table 1:** Participant anthropometric data

	Sex	Age (years)	Height (m)	Weight (kg)	BMI (kg/m^2^)
**P01**	M	24.1	1.75	65	21.2
**P02**	F	28.0	1.60	57	22.3
**P03**	F	26.4	1.71	64	21.9
**P04**	F	23.6	1.68	72	25.5
**P05**	F	21.8	1.64	70	26.0
**P06**	F	26.5	1.70	58	20.1
**P07**	F	29.4	1.61	51	19.7
**P08**	F	20.5	1.70	65	22.5
**P09**	F	26.4	1.60	60	23.4
**P10**	M	35.9	1.86	80	23.1
**P11**	M	22.5	1.73	69	23.1
**P12**	M	23.5	1.81	75	22.9
**P13**	F	24.1	1.73	65	21.7
**P14**	F	31.6	1.70	67	23.2
**P15**	M	33.2	1.85	82	24.0
**P16**	M	25.6	1.78	73	23.0
**P17**	M	29.2	1.75	75	24.5
**P18**	M	26.3	1.84	87	25.7
**P19**	M	21.7	1.75	65	21.2
**P20**	M	25.9	1.72	68	23.0
**Mean** ± **SD**	–	26.2 ± 4.0	1.73 ± 0.08	68 ± 9	22.9 ± 1.7

**Figure 2:**
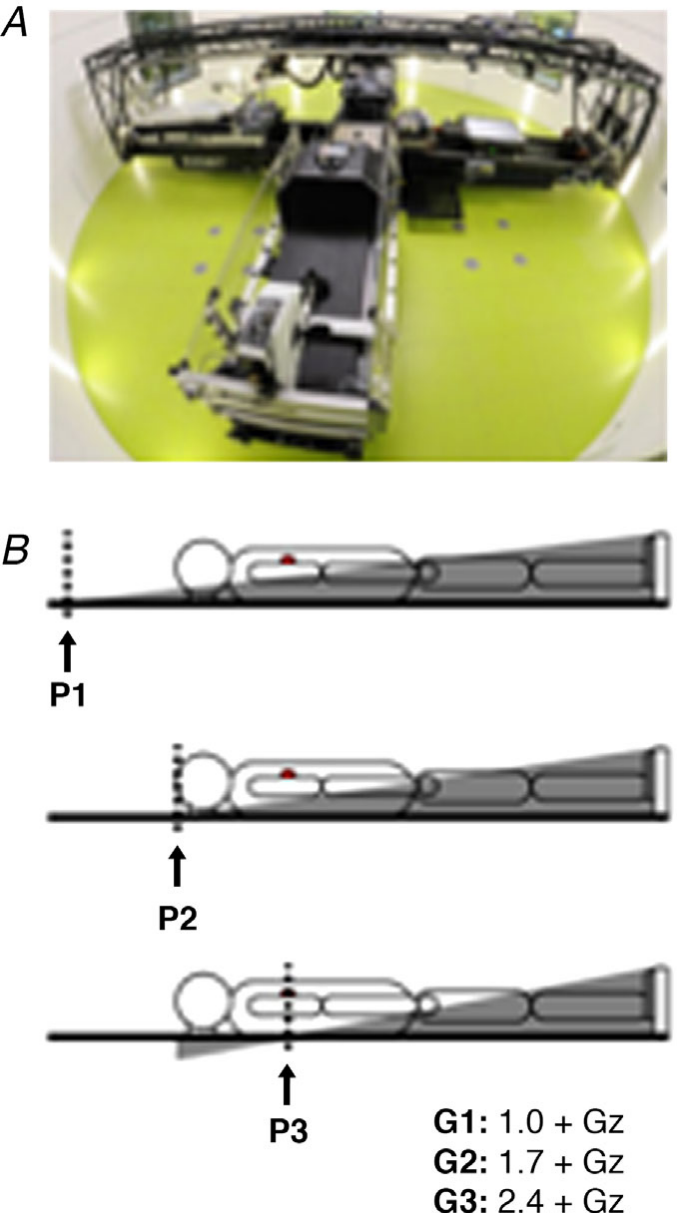
Protocol overview *A*, enviFuge short‐arm human centrifuge (SAHC) with the unique capability to alter rotational axis position and thus g‐gradient (at DLR, Cologne, Germany). *B*, three centres of rotation: P1, above the head; P2, apex of head; and P3 at heart level and three g‐levels at the feet: G1, +1.0 Gz; G2, +1.7 Gz; and G3, +2.4 Gz. *Grey shaded area*, altered g‐gradient across the body at each rotational axis position: P1, P2 and P3 (at +2.4 Gz; Table 2). [Color figure can be viewed at wileyonlinelibrary.com]

Before being enrolled in the study, all participants underwent medical screening which consisted of: clinical chemical analysis (glucose, creatinine, urea, uric acid, liver enzymes, total cholesterol, high density lipoprotein (HDL) and low density lipoprotein (LDL)); haematology (blood count); urine analysis (glucose, protein, urobilinogen, drugs); resting electrocardiogram (ECG); an exercise test to verify aerobic fitness; a standing test to assess orthostatic tolerance; and a complete medical history. Female participants had to test negative on a urine‐based pregnancy test before each session. Prior to testing, each participant was familiarized with all aspects of the study, including the methodology, personnel involved and the SAHC (Fig. 2*A*).

Participants underwent nine (following a familiarization run) passive 10 min short‐arm centrifugations on the unique :enviFuge SAHC at the German Aerospace Centre (DLR, Cologne, Germany) over two experimental sessions on separate days (2 months apart) with rest phases of at least 25 min between each of the five runs per day (Fig. 2*A*). Each participant was tested on both occasions either in the morning (09:00–13:00) or afternoon (13:00–17:00) on both occasions. Prior to each centrifugation session participants were instructed to eat a light breakfast or lunch 1 h before. For both sessions, participants were asked not to consume alcohol or caffeine in the preceding 24 h. Participants lay supine astride a saddle to support them whilst minimising muscle pump activation (including remaining silent and completely still unless experiencing significant discomfort) which was confirmed via video monitoring of the lower limbs. Each centrifuge run was performed at one of three centres of rotation (above the head, P1; apex of the head, P2; and at the heart, P3) thereby generating three differing g‐gradients across the body, standardized against the anatomical position of the heart (third intercostal space), for each participant at each of three standardized g‐levels at the feet (+1.0 Gz, +1.7 Gz and +2.4 Gz). G‐gradients are expressed as the percentage difference of g‐level at the head in relation to g‐level at the feet. At +2.4 Gz at the feet, the g‐level at the head is +0.5 at P1, 0.0 at P2 and −0.7 Gz at P3. These give rise to differences of +1.9, +2.4 and +3.1 Gz (P1, P2 and P3, respectively) and g‐gradients of +81%, P1; +100%, P2; and +130%, P3 (Table 2; and *grey shaded area*, Fig. 2*B*).

**Table 2:** Effective g‐level and g‐gradient for participants at +2.4 Gz (*n* = 15)

	G‐level at head (±Gz)	G‐level at feet (+Gz)	Delta g‐level (+Gz)	G‐gradient (%)
**Long‐arm human centrifugation (8.00 m radius)**	+1.9	+2.4	+0.5	+22
**Short‐arm human centrifugation (2.80 m radius)**	+1.1	+2.4	+1.4	+62
**P1**	+0.5	+2.4	+1.9	**+81**
**P2**	+0.0	+2.4	+2.4	**+100**
**P3**	−0.7	+2.4	+3.1	**+130**

P1, above head; P2, head apex; and P3, heart level. G‐gradient defined as the percentage difference between g‐level at the head and feet at +2.4 Gz.

**Figure 3:**
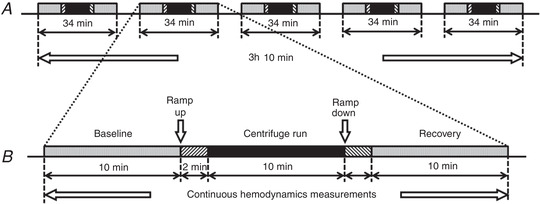
Protocol with example centrifuge profile phases *A*, protocol consisting of five randomized centrifuge profiles (34 min each), with no less than 10 min rest between them. *B*, single centrifuge profile consisting of five phases: baseline, ramp‐up, centrifuge run, ramp‐down and recovery.

Each of the two testing days consisted of five centrifuge profiles (Fig. 3*A*), with the runs after familiarization randomly assigned with an online randomizer (random.org, 2016). Each centrifuge profile was split into five phases (Fig. 3*A*) with a standardized ramp‐up and −down time (120 s) irrespective of g‐level to negate the documented effect of g‐onset time upon cardiovascular responses (Whinnery & Forster, 2013).

### Cerebral perfusion

Cerebral near‐infrared spectroscopy of the left prefrontal cortex immediately below the forehead hairline of each participant (cNIRS; PortaLite, Artinis Medical Systems, The Netherlands) was used to determine the absolute ratio of oxy‐ and deoxy‐haemoglobin. Taking into account participant frontal cranium thickness, estimated according to age and sex, the cerebral tissue saturation index (cTSI) was deduced (Lynnerup *et al*. 2005). cTSI has been proposed as a surrogate index of global cerebral perfusion (Weiss *et al*. 2005).

### Central hypovolaemia

Movement of fluid to the lower extremities, and away from the central compartment (central hypovolaemia), was quantified using strain gauge plethysmography (SGP; EC6 Strain Gauge Plethysmograph, Hokanson Inc., Bellevue, WA, USA) positioned around the calf midpoint (directly between the tibiae tuberosity and medial malleolus bony prominences) of each participant.

### Systemic cardiovascular measurements

Continuous beat‐by‐beat HR via a standard three‐lead electrocardiogram (Biopac Systems, Goleta, CA, USA) and mean arterial finger blood pressure (MAP) was recorded with a Finometer (Finapres Medical Systems, Amsterdam, The Netherlands). The Finometer finger cuff was placed around the third finger of the right hand and fixed by a sling at the level of the fourth intercostal space (i.e. heart level). Finometer BP measurements were corroborated with absolute arterial BP measurements obtained by an automated sphygmomanometer (Intellivue MMS X2, Philips, Best, The Netherlands) prior to starting each profile. Stroke volume (SV) and total peripheral resistance (TPR) was estimated from the arterial BP waveform using the Modelflow method (Leonetti *et al*. 2004) via Beatscope software (TNO‐TPD, Biomedical Instrumentation, Amsterdam, The Netherlands).

### Pre‐syncopal symptoms

For each centrifuge run all participants were classified as experiencing pre‐syncopal symptoms (PSS+) when one (or more) of the following was observed: (i) reduction of HR and BP > 15 s; (ii) nausea, paleness, dizziness; and (iii) participant requested run termination (see Goswami *et al*. 2009; Cvirn *et al*. 2012).

Participants were regularly asked during centrifugation whether they were experiencing any motion sickness symptoms, and to report any unexpected symptoms such as tunnel vision or tumbling sensations, in addition to continuous monitoring of a live video feed by an experienced physician.

### Statistical analysis

Following a pilot study of the measures used in this paper, in which comparable fluid shifts were induced via lower limb venous occlusion, accepting an error probability (α) of 0.05, power (1 ‐ β) of 0.80 with an average effect size (d) of 0.05, a power calculation, using G* Power (Erdfelder et al. 1996) yielded a participant number of 15. However, given the risk of attrition in the study, an additional 25% participants were recruited.

All continuously recorded data were inspected in 10 s frames and artefacts removed with a Matlab (R2015a, The Mathworks Inc.) function using the following criteria: (i) physiologically plausible limits; and (ii) maximal percentage of change in relation to the standard deviation of the signal. Data were resampled at 4 Hz (piecewise cubic spline interpolation) as long as >95% of data were valid (<5% artefact removal rate) yielding 15 complete sets of data for further analysis. Summary data are presented as means ± standard deviation (SD) unless otherwise stated. Mean 2 min changes (delta) from the mean of the final 5 min of the 10 min baseline immediately before each corresponding centrifuge run were analysed.

Data are presented as mean 2 min changes (delta) from the mean of the final 5 min of the 10 min baseline immediately before each corresponding centrifuge run.

R (R Core Team, 2012) and *lme4* (Bates *et al*. 2012) were used to perform linear mixed effect analyses of the relationship between each cardiovascular parameter and the time course of each centrifuge run. Time, position and g‐level at the feet (with two‐ and three‐way interactions) were entered into the model with intercepts for each participant as a random effect. Visual inspection of residual plots did not reveal any obvious deviations from homoscedasticity or residual normality. *P* values were obtained by likelihood ratio tests of the full model with the effect (time, position and g‐level at the feet) in question, against the model without that effect.

To reduce complexity of the models, secondary analysis was performed with data both averaged for each 2 min increment of centrifugation and compared for differences arising due to position at that moment. To evaluate the differences induced (e.g. P1 *vs*. P3), one‐way analyses of variance were conducted, followed by *post hoc* tests (Tukey's honestly significant difference) to determine effect location (i.e. time, and thus phase, in centrifugation). Chi‐squared testing was employed to evaluate the frequency of PSS+ classification during runs at each position across all g‐levels (+1.0, +1.7 and +2.4 Gz).

## Results

### G‐gradient, g‐level and duration

Significant changes were observed in all measured cardiovascular variables except MAP where there was a strong trend for time (i.e. centrifugation) (Table 3; *P* < 0.05). All parameters were significantly affected by g‐level, and all but CO were affected by position (Table 4; *P* < 0.05). As there was a significant effect of g‐level on all parameters, for clarity the figures only show P3 and P1 +2.4 Gz data as P2 responses were consistently around the midpoint between those of P1 and P3 across the entire centrifuge run (ramp‐up, centrifugation, ramp‐down and recovery).

**Table 3:** Effects (±SD) of cardiovascular variables as a function of time, position and g‐level at feet (fixed effects) based on linear mixed effect testing (*n* = 15). ^*^ *P* < 0.05

	(Intercept)			Time (s)			Position (head level)			Foot g‐level (+Gz_feet_)		
	Effect ± SD	*F* value	*P* value	Effect ± SD	*F* value	*P* value	Effect ± SD	*F* value	*P* value	Effect ± SD	*F* value	*P* value
cTSI (%)	2.42 ± 1.28	18.8	<0.001	−0.002 ± 0.004	6.55	<0.05	−0.66 ± 0.465	1194	<0.001	−1.46 ± 0.620	2452	<0.001
CC (mm)	0.102 ± 1.67	32.0	<0.001	0.001 ± 0.004	1793	<0.001	−0.112 ± 0.445	1035	<0.001	0.379 ± 0.600	2938	<0.001
HR (bpm)	−8.98 ± 6.86	54.0	<0.001	0.009 ± 0.016	658	<0.05	−2.63 ± 2.32	6365	<0.001	3.53 ± 3.10	13038	<0.001
SV (ml)	17.40 ± 8.13	409	<0.001	−0.015 ± 0.019	78.6	<0.001	−9.70 ± 3.29	1949	<0.001	−16.6 ± 4.45	3037	<0.001
TPR (dyn.s.cm^−5^)	−239.2 ± 198.1	136	<0.001	0.241 ± 0.523	44.1	<0.001	199.0 ± 80.9	678	<0.001	222.7 ± 108.8	14.6	<0.001
MAP (mmHg)	−9.56 ± 5.38	3.65	0.056	0.011 ± 0.012	3.58	0.058	3.13 ± 2.05	3537	<0.001	1.48 ± 2.75	196	<0.001
CO (l min^−1^)	0.382 ± 0.682	114	<0.001	−0.001 ±0.001	6.80	<0.01	−0.612 ± 0.279	0.009	0.924	−0.739 ± 0.376	15.0	<0.001

Time (s), from the start of the centrifuge phase; position expressed as a multiple of the distance from heart level to the apex of the head (in relation to the rotational axis); +Gz_feet_, g‐level experienced during each profile at foot level.

cTSI, cerebral tissue saturation index; CC, calf circumference – strain gauge plethysmography; HR, heart rate; SV, stroke volume; TPR, total peripheral resistance; MAP, mean arterial pressure; CO, cardiac output.

**Table 4:** Delta response (±SD) of cardiovascular variables as a function of position (P1–3) at +2.4 Gz in the final minute of centrifugation (minute 10) (*n* = 15) based on P1 *vs*. P(*n*), via Tukey's honestly significant difference. ^*^ *P* < 0.05

	Tenth minute @ +2.4 Gz		
	P1	P2	P3
ΔcTSI (%)	−2.85 ± 1.86	−1.88 ± 1.43^*^	−0.95 ± 1.32^*^
ΔCC (mm)	5.46 ± 2.09	4.11 ± 2.44^*^	2.23 ± 1.63^*^
ΔHR (bpm)	50.0 ± 15.5	24.0 ± 11.62^*^	8.0 ± 7.75^*^
ΔSV (ml)	−37.7 ± 12.0	−27.2 ± 9.30^*^	−19.4 ± 6.58^*^
ΔTPR (dyn.s.cm^−5^)	204 ± 290	223 ± 205	169 ± 136^*^
ΔMAP (mmHg)	4.43 ± 18.10	4.60 ± 9.10	−3.87 ± 3.25^*^
ΔCO (l min^−1^)	−0.64 ± 1.32	−0.59 ± 0.77	−0.69 ± 0.39

cTSI, cerebral tissue saturation index; CC, calf circumference – strain gauge plethysmography; HR, heart rate; SV, stroke volume; TPR, total peripheral resistance; MAP, mean arterial pressure; CO, cardiac output.

### Cerebral perfusion and central hypovolaemia

**Figure 4:**
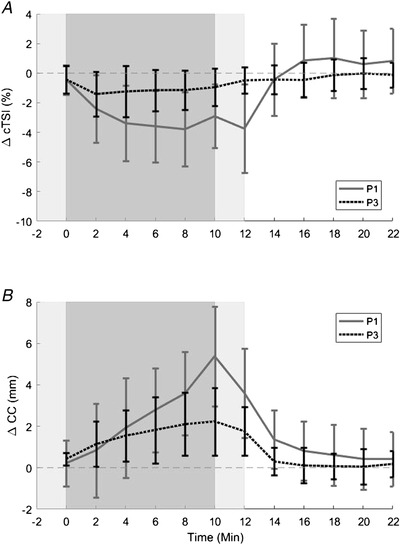
Mean (±SD) delta cerebral tissue saturation index (ΔcTSI) and calf circumference (ΔCC) in P1 *vs*. P3 at +2.4 Gz Shading: *light grey* (‐2 to 0 min and 10 to 12 min) centrifuge ramp‐up and ramp‐down phases respectively; *dark grey* (0 to 10 min) centrifuge run phase at +2.4 Gz; *white* (12 to 22 min) recovery phase. Continuous line: rotational axis position above head (P1) with +2.4 Gz; dotted line: rotational axis position at heart level (P3) with +2.4 Gz. Mean delta (from 5 min mean of respective baseline immediately before) during the ramp‐up, run, ramp‐down and recovery phases of centrifugation in P1 *vs*. P3 at 2.4 +Gz: *A*, mean delta cerebral tissue saturation index (ΔcTSI); and *B*, mean delta calf circumference (ΔCC). Positions were compared via Tukey's honesty significant difference testing (*n* = 15); ΔcTSI. Position: *F*_8,171_ = 1194; ΔCC, position: *F*_8,171_ = 1035, ^*^ *P* < 0.05.

Delta cerebral perfusion (ΔcTSI) reductions were significantly attenuated from the fourth minute until the end of the ramp‐down phase between P1 and P3 at +2.4 Gz (Fig. 4*A*). Conversely, delta calf circumference (ΔCC) increases were significantly attenuated between P1 and P3 in the final 2 min of centrifugation, the ramp‐down phase and the first 2 min of recovery (Fig. 4*B*).

### Attenuated cardiovascular responses

**Figure 5:**
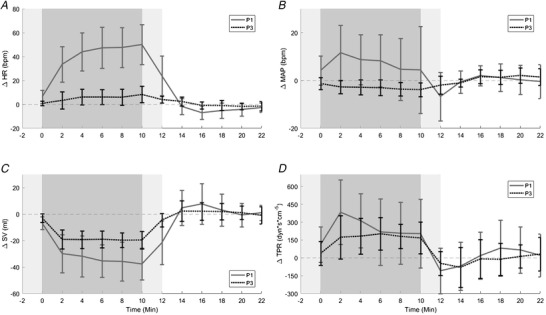
Mean (± SD) delta heart rate (ΔHR), mean arterial pressure (ΔMAP), stroke volume (ΔSV) and total peripheral resistance (ΔTPR) in P1 *vs*. P3 at 2.4 +Gz Shading: *light grey* (‐2 to 0 min and 10 to 12 min) centrifuge ramp‐up and ramp‐down phases respectively; *dark grey* (0 to 10 min) centrifuge run phase at +2.4 Gz; *white* (12 to 22 min) recovery phase. Continuous line: rotational axis position above head (P1) with +2.4 Gz; dotted line: rotational axis position at heart level (P3) with +2.4 Gz. Mean delta of all participants (from 5 min mean of respective baseline immediately before) during the ramp‐up, run, ramp‐down and recovery phases of centrifugation in P1 *vs*.P3 at +2.4 Gz: *A*, mean delta heart rate (ΔHR); *B*, mean delta mean arterial pressure (ΔMAP); *C*, mean delta stroke volume (ΔSV); and *D*, total peripheral resistance (ΔTPR). Positions were compared via Tukey's honesty significant difference testing (*n* = 15); ΔHR, position: *F*_8,171_ = 1194; ΔSV, position: *F*_8,171_ = 1035; ΔTPR, position: *F*_8,171_ = 1035. ^*^ *P* < 0.05.

Moving RAP towards the body (P3) and away from ‘classical centrifugation’ (P1) resulted in significant attenuation of HR (Fig. 5*A*) and MAP (Fig. 5*B*) increases, and SV (Fig. 5*C*) decreases in response to centrifugation. However, attenuation of early phase TPR increments (observed in P1) was significant only in the second and fourth minutes (Fig. 5*B*).

Delta heart rate (ΔHR) acceleration was greatest in the final minute of centrifugation in both P1 and P3; albeit significantly less in the latter (ΔHR: 50 ± 4 bpm to 8 ± 2 bpm, *P* < 0.05; Fig. 5*A*). Delta mean arterial pressure (ΔMAP) remained essentially unchanged during P3 centrifugation (Fig. 5*B*) in contrast to during P1 where substantial increments were observed, peaking at the second minute before progressively reducing through the remaining centrifugation run (Fig. 5*B*). Thus, ΔMAP was significantly (*P* < 0.05) higher during the entire centrifugation phase in P1 *vs*. P3, and then lower in P1 (*vs*. P3) (7.05 ± 2.92 mmHg to −1.94 ± 0.98 mmHg) in the first minute following ramp‐down as no rebound was evident (P1 *vs*. P3, ΔMAP: −7.05 ± 2.92 mmHg to −1.94 ± 0.98 mmHg, *P* < 0.05; Fig. 5*B*). Delta stroke volume (ΔSV) reductions were significantly lower in P1 from the second minute of centrifugation onwards until the end (Fig. 5*C*). Delta total peripheral resistance (ΔTPR) increments were unaffected by position, except from the second and fourth minutes, where it was significantly lower in P3 (Fig. 5*D*).

### Increased g‐tolerance

**Figure 6:**
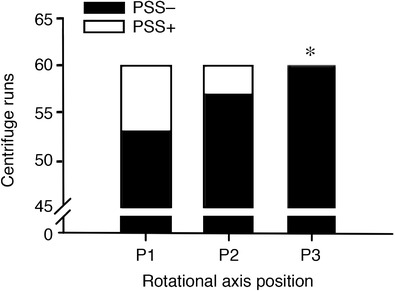
G‐tolerance in each position across all g‐levels (+1.0, +1.7 and +2.4 Gz) All participants were exposed to nine centrifuge profiles giving rise to 180 separate centrifuge runs, consisting of 60 in each position: P1, rotational axis position (RAP) above head; P2, RAP head apex; P3, RAP heart level. Participants classified as having experienced pre‐syncopal symptoms (PSS**+**, white) or not experiencing PSS (PSS‐, black). Levels were compared via Chi‐squared testing (*n* = 15); c^2^(1, *n* = 15) = 7.43, ^*^ *P* < 0.05.

The proportion of participants classified as experiencing PSS+ was significantly (*P* < 0.05) lower at P3 *vs*. P1 across the +Gz levels (Fig. 6). The proportion in P2 also tended to be lower than P1, albeit not significant.

### Unsupported cerebral perfusion

**Figure 7:**
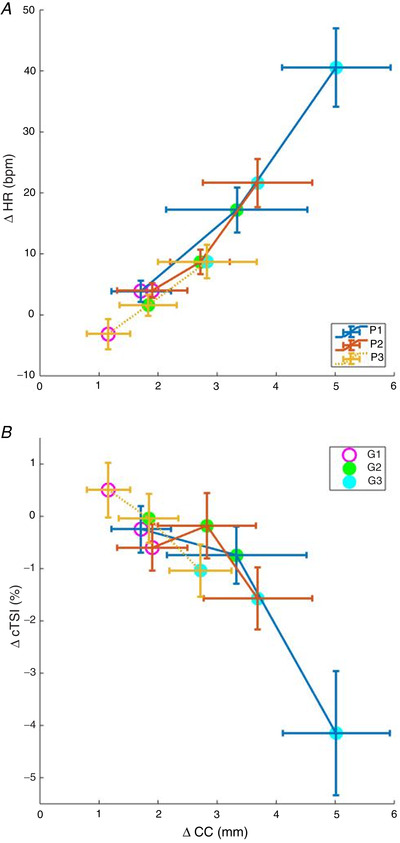
Relationship between mean delta heart rate (ΔHR) and cerebral tissue saturation index (ΔcTSI) *vs*. calf circumference (ΔCC) during centrifugation at all g‐levels and positions Black line: rotational axis position above head (P1); grey line: rotational axis position at apex of head (P2); dotted line: rotational axis position at heart level (P3); white circle: g‐level 1.0 +Gz at feet (G1); light grey circle: g‐level 1.7 +Gz at feet (G2); dark grey circle: g‐level 2.4 +Gz at feet (G3). *A*, mean ± SD delta heart rate (ΔHR) and *B*, mean delta cerebral tissue saturation index (ΔcTSI) against delta calf circumference (ΔCC) obtained from the last minute of each centrifuge run. Relationships between parameters were assessed via Pearson correlation (*n* = 15). ^*^ *P* < 0.05.

A strong positive linear relationship was observed between ΔCC and ΔHR with increasing +Gz, independent of position (Fig. 7*A*). In contrast, ΔCC *vs*. ΔcTSI demonstrated a strong negative relationship with +Gz, independent of position (Fig. 7*B*).

## Discussion

The purpose of the study was to investigate (for the first time) the effect of three standardised RAPs and the head‐to‐heart and heart‐to‐foot hydrostatic component (g‐gradient) of blood pressure upon the cardiovascular responses, peripheral blood displacement (i.e. central hypovolaemia), cerebral perfusion and g‐tolerance, in addition to the relationship between peripheral blood displacement, and both HR and cerebral perfusion with respect to g and RAP. Specifically, we sought: (i) to determine physiological differences to positive and negative heart‐to‐head g‐gradients by measuring cerebral perfusion, systemic cardiovascular responses and the emergence of pre‐syncopal symptoms indicative of g‐intolerance; and (ii) provide a better insight for future artificial gravity research by characterizing the third variable in human centrifugation studies (namely rotational axis placement in addition to duration and magnitude of exposure). The main findings were that a reduced or negative heart‐to‐head g‐gradient significantly attenuated the reduction in cerebral perfusion and central hypovolaemia, the acceleration of HR and indicators of g‐intolerance.

### Cardiovascular receptors, HIPs and gravitational fields

In P1, the fluid shifts and overall cardiovascular loading is comparable to that of upright standing (Goswami *et al*. 2015*a*). In contrast, increasing footward g‐load in P3 offers a unique situation: minimal hydrostatic signal to the cardiopulmonary and aortic baroreceptors (since they sit on, or very close to, the RAP) whilst the carotid receptors experience positive g‐gradient loading, albeit relatively small (e.g. approximately about 5 mmHg in Fig. 1) given that there is a distance of 15–20 cm to the heart.

In fact, pressure sensitive receptor stimulation consists of two parts: direct hydrostatic effects due to the position within the vascular compartment (arterial, venous); and secondary blood shifts due to acceleration gradients throughout the entire system. Even if a baroreceptor remains unaffected at the onset of centrifugation by being located at the centre of rotation, the g‐gradient will redistribute blood causing local pressure changes, which will initiate secondary reflex cardiovascular responses. The effects of altered pressure gradients when RAP is changed are demonstrated in Fig. 1. The diversion from changes under normal orthostatic standing challenge to those observed during centrifugation is presented in Table 5. However, the positions of arterial and venous HIPs during centrifugation, and their dependence on absolute g‐force and body positioning within the respective g‐field, has yet to be determined.

**Table 5:** Estimated hydrostatic blood pressure differences between the heart and cardiovascular points on the body based on the average body length (1.73 m) in this study with 2.4 *g* at foot level. Anthropometric positions are approximate and may vary from person to person

	Top of head	Eye level	Carotid baroreceptor	Aortic baroreceptor	Heart	Foot
Centrifuge						
P1						
r_p_ (m)	0.49	0.59	0.70	0.92	0.97	2.22
ΔP_heart_ (mmHg)	−29.7	−24.8	−18.9	−4.3	0	166
P2						
r_p_ (m)	0	0.11	0.22	0.43	0.49	1.73
ΔP_heart_ (mmHg)	−12.7	−12.0	−10.2	−2.7	0	148
P3						
r_p_ (m)	0.49	0.38	0.27	0.05	0	1.24
ΔP_heart_ (mmHg)	17.6	10.7	5.4	0.2	0	115
Standing						
h (m)	1.73	1.62	1.51	1.30	1.24	0
ΔP_heart_ (mmHg)	−37.6	−29.2	−20.9	−4.2	0	96.0

Centrifuge: ∆P_heart_: pressure difference between body point and heart level {= ρ k_BP_gfoot/h_foot_($r_{p}^{2}-r_{\mathrm{heart}}^{2}$), ρ: density of blood 1050 kg/m^3^, k_BP_: 0.0075 (conversion factor: Pa to mmHg), r_p_: distance from centre of centrifuge}.

Standing: ∆P_heart_: pressure difference between body point and heart level {= ρk_BP_gΔh, ρ: density of blood, k_BP_: 0.0075 (conversion factor Pa to mmHg), Δh: vertical distance between the height of the heart and height from the feet (h)}.

### Initial response

Delta calf circumference was unchanged by position in the initial stages of centrifugation (up until minute eight) whilst ΔcTSI was significantly lower at +2.4 Gz in P1 than P3 from the fourth minute onwards. This finding suggests that cerebral perfusion was attenuated earlier than significant peripheral fluid shifts indicated by ΔCC, presumably due to a varied peripheral vascular response to early orthostatic stimulation (Watenpaugh *et al*. 2004). By the final minute of centrifugation, a concurrent increase in ΔCC and ΔcTSI decrease was observed, suggestive that this simple measure of peripheral fluid shifts may have utility as a proxy of cerebral haemodynamics during sustained SAHC.

### Central hypovolaemia

Delta calf circumference, ΔHR, ΔTPR and mean arterial pressure (ΔMAP) all increased as a function of g‐level, as hypothesized based on previous findings (Goswami *et al*. 2015*a*). Similarly, ΔcTSI and ΔSV decreased in line with the literature (Smith *et al*. 2013). However, we also observed an almost perfect linear relationship between ΔCC and ΔHR, irrespective of position and g‐level. The strength of this relationship was unexpected and suggests a consequential systemic response to calf pooling, likely caused by volume shifts, and thus decreased cardiac return (pre‐load) and filling pressure (Rowell *et al*. 1996). Such a relationship is similar to that presented by Hachiya *et al*. (2010) during lower body negative pressure where differences in tolerant (rightward shift) and intolerant (leftward shift) HR responses, as a function of calf circumference, were explained by splanchnic region constriction.

Whilst our study showed an increase in ΔCC, the effect upon splanchnic blood volume was not determined. In fact, splanchnic vasoconstriction has been observed in response to orthostatic challenge to maintain venous return (Hinghofer‐Szalkay *et al*. 2008; Blaber *et al*. 2013). Thus, future studies should evaluate the relationship between calf circumference and splanchnic blood volume changes to SAHC.

### Cerebral perfusion

It was hypothesized that cerebral perfusion support would be observed at P3. However, this was not the case. One possible explanation is that increased arterial headward pressure results in venous congestion, thereby limiting oxygenation. Further work is required to confirm this, and to determine the relationship between jugular vein congestion and cerebral haemodynamics with g‐gradients. Should venous congestion during in‐body centrifugation be confirmed, it may provide a valuable methodology with which to investigate its role in spaceflight‐acquired neuro‐ocular syndrome observed in some astronauts (Zhang & Hargens, 2014).

### Gravitational gradient

The ‘total gravitational exposure’, i.e. g‐gradient across the body (and thus pressure sensors), changes with each position despite a comparable +Gz level at the feet (Fig. 1). It is known that the hydrostatic gradient is of critical importance in determining an orthostatic challenge, and the response to it, particularly during passive stress (Hinghofer‐Szalkay, 2011). Thus, resultant modification of aortic and carotid baroreceptor feedback may explain the fact that the cardiovascular response in P3 at a high +Gz level was similar to that elicited at a low +Gz level in P1. Furthermore, the g‐gradient of P3 was shown to increase g‐tolerance – suggestive of preservation of cerebral oxygenation – a key limiting factor for centrifuge training.

Significant attenuations of g‐induced changes in cerebral perfusion, central hypovolaemia and systemic cardiovascular response occurred at P3, the most striking of which was ΔHR. Interestingly, it has been postulated that the hydrostatic gradient between the carotid and aortic baroreceptors is a potent determinant of HR responses to orthostatic challenge (Ferguson *et al*. 1985). In the present study, at P3 the hydrostatic gradient was reversed, suggesting (in isolation) that bradycardia should ensue. However, ΔHR at P3 represented a ‘dampened’ increase, likely due to the removal of central blood volume having an opposing effect on ΔHR. This working hypothesis could be tested by prevention of the fluid shift during centrifugation via the use of anti‐g trousers (Gray et al. 1969).

### Varying g‐gradients during AG: advantages over current countermeasures

Current spaceflight countermeasures on board the International Space Station attempt to ameliorate the effects of gravitational unloading through an extensive daily exercise programme (Petersen *et al*. 2016). Training consists of treadmill running, cycle ergometry and resistive exercise, which together have some (albeit variable) effectiveness at reducing loss of muscle mass (Trappe *et al*. 2009), bone density (Shackelford *et al*. 2004) and cardiorespiratory function (Loehr *et al*. 2011). However, despite such current countermeasures, typically 30–50% of all returning astronauts demonstrate OI on return (Moore *et al*. 1996; Blaber *et al*. 2011). It is postulated that high OI incidence arises from the failure of current countermeasures to counteract headward fluid shifts; thus combining exercise with AG may prove more effective.

## Limitations

An obvious criticism is the use of the cerebral tissue saturation index as a proxy for cerebral blood delivery. This approach has been validated, but it may have been affected by changes in skin thickness (secondary) due to fluid shifts and of course the reduction in flow may have negatively affected NIRS signal quality (and thus validity). Furthermore, this study did not measure local pressure or volume changes during centrifugation; pressure changes were calculated from referring g‐profiles. Speculation regarding pressure receptor stimulation and arterial/venous HIP locations has been inferred based upon studies of the cardiovascular responses to postural changes (Hinghofer‐Szalkay *et al*, 2011; Patel *et al*. 2016). More research is needed to obtain pressure/volume recordings during centrifugation in order to differentiate the passive haemodynamic responses from those generated by resultant autonomic regulation.

## Conclusions

Our data confirm our hypothesis that movement of RAP towards the heart (reduced g‐gradient) independent of foot‐level Gz, reduced the orthostatic challenge leading to improved g‐tolerance. However, the role of peripheral blood displacement remains unclear. Our results suggest that RAP, and thus g‐gradient, warrants further investigation, specifically the effect of differential baroreceptor feedback (i.e. aortic–carotid g‐gradient) as reduced cardiac filling may effect haemodynamic regulation during and immediately following SAHC.

Our results demonstrate that SAHC g‐gradient is a critical factor in determining the haemodynamic responses to centrifugation in addition to g‐level magnitude and duration. However, moving the axis of rotation to the level of the heart probably decreases the potential orthostatic training benefits of SAHC in microgravity, although this, and its effect on the efficacy of AG on other physiological systems, also warrants further investigation.

## Additional information

### Competing interests

No conflicts of interest, financial or otherwise, are declared by the authors.

### Author contributions

CL performed the experiments; CL and JR analysed the data; CL, DAG, NG, EM and JR conceived and designed the research; CL, DAG, AB, HHS, NG interpreted the results of the experiments; CL, DAG, AB, HHS, EM, JR and NG edited and revised the manuscript; CL,DAG, AB, HHS, EM, JR and NG approved the final version of the manuscript.

### Funding

CL received a Helmholtz Space Life Sciences Research School (SpaceLife) PhD scholarship. SpaceLife was funded in equal parts by the Helmholtz Association (grant no.: VH‐KO‐300) and the German Aerospace Centre (DLR: internal cost object 2547 121).

## Supporting information


**Statistical Summary Document**
