# Supplementary material for: Effect of novel short‐arm human centrifugation‐induced gravitational gradients upon cardiovascular responses, cerebral perfusion and g‐tolerance
Source: J Physiol. 2020 Aug 19;598(19):4237–49. doi: 10.1113/JP273615 (PMC7589294; doi:10.1113/JP273615)
Supplement: Supplementary file 1 — Statistical Summary Document [file TJP-598-4237-s001.doc]

**Manuscript Title:** Effect of novel short-arm human centrifugation induced gravitational gradients upon cardiovascular responses, cerebral perfusion and g-tolerance

**Authors:** Charles Laing, David A. Green, Edwin Mulder, Helmut Hinghofer-Szalkay, Andrew P. Blaber, Joern Rittweger, Nandu Goswami

**Animal model used, if applicable:** N/A

**Underlying hypothesis:**

Movement of the Rotational Axis Position (RAP) from (the conventional position) above the head towards the heart shall reduce central hypovolemia, limit cardiovascular responses, aid cerebral perfusion, and thus promote g-tolerance.

**Definitions of ‘n’:**

Question 1: n = number of participants.

Question 2: n = number of participants.

Question 3: n = number of participants.

Question 3: n = number of participants.

Statistical summary table:

| Experimental question number* | Finding/ conclusion | Experimental location/ variable  e.g. cortex vs cerebellum or genotype | Mean value  (or other summary statistic) | SD | n (value) | P** | Units | Data comparisons  e.g. WT vs KO | Statistical test | Any other variable  e.g. subjects’ age or sex | Figure/table in which data are presented | Comments  e.g. observation |
| --- | --- | --- | --- | --- | --- | --- | --- | --- | --- | --- | --- | --- |
| 1. Movement of the RAP from (the conventional position) above the head towards the heart may reduce central hypovolemia, limit cardiovascular responses, aid cerebral perfusion | Cerebral tissue saturation index, calf circumference and heart rate responses will be reduced thereby promoting g-tolerance | cTSI | -0.66 | 0.47 | 15 | < 0.001 | % | Effect of position (P1, P2, P3) | Linear Mixed Effect Analysis (LME) |  | Table. 3 |  |
|  |  | CC | -0.112 | 0.445 | 15 | < 0.001 | mm | Effect of position (P1, P2, P3) | LME |  | Table. 3 |  |
|  |  | HR | -2.63 | 2.32 | 15 | < 0.001 | bpm | Effect of position (P1, P2, P3) | LME |  | Table. 3 |  |
|  |  | SV | -9.70 | 3.29 | 15 | < 0.001 | mL | Effect of position (P1, P2, P3) | LME |  | Table. 3 |  |
|  |  | TPR | 199.0 | 80.9 | 15 | < 0.001 | Dyn.sc.m^-3^ | Effect of position (P1, P2, P3) | LME |  | Table. 3 |  |
|  |  | MAP | 3.13 | 2.05 | 15 | < 0.001 | mmHg | Effect of position (P1, P2, P3) | LME |  | Table. 3 |  |
|  |  | CO | -0.612 | 0.279 | 15 | 0.924 | L.min^-1^ | Effect of position (P1, P2, P3) | LME |  | Table. 3 |  |
| 2. Movement of the RAP from (the conventional position) above the head towards the heart may reduce central hypovolemia, limit cardiovascular responses, aid cerebral perfusion (P1 vs. P3) | Cerebral tissue saturation index, calf circumference and heart rate responses will be reduced thereby promoting g-tolerance | ΔcTSI (P1) | -2.85 | 1.86 | 15 | - | % | Effect of position (P1 vs. P3) | Tukey’s Honesty Significant Difference |  | Table. 4 / Figure. 4a |  |
|  |  | ΔCC (P1) | 5.46 | 2.09 | 15 | - | mm | Effect of position (P1 vs. P3) | Tukey’s Honesty Significant Difference |  | Table. 4 / Figure. 4b |  |
|  |  | ΔHR (P1) | 50 | 15.5 | 15 | - | bpm | Effect of position (P1 vs. P3) | Tukey’s Honesty Significant Difference |  | Table. 4 |  |
|  |  | ΔSV (P1) | -37.7 | 12.0 | 15 | - | mL | Effect of position (P1 vs. P3) | Tukey’s Honesty Significant Difference |  | Table. 4 |  |
|  |  | ΔTPR (P1) | 204 | 290 | 15 | - | Dyn.sc.m^-3^ | Effect of position (P1 vs. P3) | Tukey’s Honesty Significant Difference |  | Table. 4 |  |
|  |  | ΔMAP (P1) | 4.43 | 18.10 | 15 | - | mmHg | Effect of position (P1 vs. P3) | Tukey’s Honesty Significant Difference |  | Table. 4 |  |
|  |  | ΔCO (P1) | -0.64 | 1.32 | 15 | - | L.min^-1^ | Effect of position (P1 vs. P3) | Tukey’s Honesty Significant Difference |  | Table. 4 |  |
|  |  | ΔcTSI (P3) | -0.95 | 1.32 | 15 | p < 0.05 | % | Effect of position (P1 vs. P3) | Tukey’s Honesty Significant Difference |  | Table. 4 / Figure. 4a |  |
|  |  | ΔCC (P3) | 2.23 | 1.63 | 15 | p < 0.05 | mm | Effect of position (P1 vs. P3) | Tukey’s Honesty Significant Difference |  | Table. 4 / Figure. 4b |  |
|  |  | ΔHR (P3) | 8.0 | 7.8 | 15 | p < 0.05 | bpm | Effect of position (P1 vs. P3) | Tukey’s Honesty Significant Difference |  | Table. 4 |  |
|  |  | ΔSV (P3) | -19.4 | 6.6 | 15 | p < 0.05 | mL | Effect of position (P1 vs. P3) | Tukey’s Honesty Significant Difference |  | Table. 4 |  |
|  |  | ΔTPR (P3) | 169 | 136 | 15 | p < 0.05 | Dyn.sc.m^-3^ | Effect of position (P1 vs. P3) | Tukey’s Honesty Significant Difference |  | Table. 4 |  |
|  |  | ΔMAP (P3) | -3.87 | 3.25 | 15 | p < 0.05 | mmHg | Effect of position (P1 vs. P3) | Tukey’s Honesty Significant Difference |  | Table. 4 |  |
|  |  | ΔCO (P3) | -0.69 | 0.39 | 15 | p > 0.05 | L.min^-1^ | Effect of position (P1 vs. P3) | Tukey’s Honesty Significant Difference |  | Table. 4 |  |
| 3. Movement of the RAP from (the conventional position) above the head towards the heart may promote g-tolerance (lower PSS+ frequency) | Movement of the RAP from (the conventional position) above the head towards the heart promoted g-tolerance | PSS+ (P1) | 53 out of 60 | - | 15 | - |  |  | - |  | Figure. 6 |  |
|  |  | PSS+ (P2) | 57 out of 60 | - | 15 |  |  | Effect of position (P1 vs. P2) | Chi-Squared |  | Figure. 6 |  |
|  |  | PSS+ (P3) | 60 out of 60 | - | 15 |  |  | Effect of position (P1 vs. P3) | Chi-Squared |  | Figure. 6 |  |
| 4. Relationship between ΔCC vs. ΔHR and Relationship between ΔCC vs. ΔHR acros s positions and g levels | A strong positive linear relationship between ΔCC and ΔHR with increasing +Gz, independent of position. ΔCC vs. ΔcTSI demonstrated a strong negative relationship with +Gz, independent of position | ΔCC vs. ΔHR | - | - | 15 |  | p < 0.05 | Relationship between ΔCC vs. ΔHR | Pearson Correlation |  | Figure. 7a |  |
|  |  | ΔCC vs. ΔTSI | - | - | 15 |  | p > 0.05 | Relationship between ΔCC vs. ΔTSI | Pearson Correlation |  | Figure. 7b |  |

*You may use multiple lines for the same question to indicate multiple comparisons

** Authors may wish to make the text bold where p is considered significant against a stated confidence limit
